# Supplementary material for: Gastrostomy and congenital anomalies: a European population-based study
Source: BMJ Paediatr Open. 2022 Jun 24;6(1):e001526. doi: 10.1136/bmjpo-2022-001526 (PMC9234789; doi:10.1136/bmjpo-2022-001526)
Supplement: Author's manuscript [file bmjpo-2022-001526.draft_revisions.pdf]

BMJ Paediatrics Open

BMJ Paediatrics Open is committed to open peer review. As part of this commitment we make the peer review history of every article we publish publicly available.

When an article is published we post the peer reviewers' comments and the authors' responses online. We also post the versions of the paper that were used during peer review. These are the versions that the peer review comments apply to.

The versions of the paper that follow are the versions that were submitted during the peer review process. They are not the versions of record or the final published versions. They should not be cited or distributed as the published version of this manuscript.

BMJ Paediatrics Open is an open access journal and the full, final, typeset and author-corrected version of record of the manuscript is available on our site with no access controls, subscription charges or pay-per-view fees (http://bmjpaedsopen.bmj.com).

If you have any questions on BMJ Paediatrics Open's open peer review process please email info.bmjpo@bmj.com

BMJ Paediatrics Open

Gastrostomy and congenital anomalies - a European population-based study

Journal:	*BMJ Paediatrics Open*
Manuscript ID	bmjpo-2022-001526
Article Type:	Original research
Date Submitted by the Author:	26-Apr-2022

Complete List of Authors:	Garne, Ester; Lillebaelt Hospital - University Hospital of Southern Denmark, Department of Paediatrics and Adolescent Medicine<br>Tan, Joachim; Population Health Research Institute, St George's, University of London,<br>Loane, Maria; Ulster University, Centre for Maternal, Fetal and Infant Research, INHR<br>Baldacci, Silvia; Institute of Clinical Physiology National Research Council,<br>Ballardini, Elisa; University of Ferrara, Neonatal Intensive Care Unit, Paediatric Section, IMER Registry (Emilia Romagna Registry of Birth Defects), Dep. of Medical Sciences<br>Brigden, Joanne; Population Health Research Institute, St George's, University of London<br>Cavero-Carbonell, Clara; Foundation for the Promotion of Health and Biomedical Research in the Valencian Region FISABIO, Rare Diseases Research Unit<br>García-Villodre, Laura; Foundation for the Promotion of Health and Biomedical Research in the Valencian Region FISABIO, Rare Diseases Research Unit<br>Gissler, Mika; National Institute for Health and Welfare,<br>given, joanne; Ulster University, Centre for Maternal, Fetal and Infant Research, INHR<br>Heino, Anna; Finnish Institute for Health and Welfare<br>Jordan, Sue; Swansea University, College of Human and Health Sciences<br>Limb, Elizabeth; Population Health Research Institute, St George's, University of London<br>Neville, Amanda; Registro IMER, Azienda Ospedaliero - Universitaria di Ferrara<br>Rissmann, Anke; Malformation Monitoring Centre Saxony-Anhalt, Medical Faculty Otto-von-Guericke University,<br>Santoro, Michele; Unit of Epidemiology of Rare diseases and Congenital anomalies, Institute of Clinical Physiology, National Research Council<br>Scanlon, Ieuan; Faculty of Medicine, Health and Life Sciences, Swansea University<br>Urhoj, Stine; University of Copenhagen, Department of Public Health; Hospital Lillebaelt Kolding, Paediatric Department<br>Wellesley, Diana; Princess Anne Hospital, Wessex Clinical Genetics Service<br>Morris, Joan; St George's, University of London, Population Health

	Research Institute
Keywords:	Epidemiology

SCHOLARONE™ Manuscripts

I, the Submitting Author has the right to grant and does grant on behalf of all authors of the Work (as defined in the below author licence), an exclusive licence and/or a non-exclusive licence for contributions from authors who are: i) UK Crown employees; ii) where BMJ has agreed a CC-BY licence shall apply, and/or iii) in accordance with the terms applicable for US Federal Government officers or employees acting as part of their official duties; on a worldwide, perpetual, irrevocable, royalty-free basis to BMJ Publishing Group Ltd ("BMJ") its licensees and where the relevant Journal is co-owned by BMJ to the co-owners of the Journal, to publish the Work in this journal and any other BMJ products and to exploit all rights, as set out in our licence.

The Submitting Author accepts and understands that any supply made under these terms is made by BMJ to the Submitting Author unless you are acting as an employee on behalf of your employer or a postgraduate student of an affiliated institution which is paying any applicable article publishing charge ("APC") for Open Access articles. Where the Submitting Author wishes to make the Work available on an Open Access basis (and intends to pay the relevant APC), the terms of reuse of such Open Access shall be governed by a Creative Commons licence – details of these licences and which Creative Commons licence will apply to this Work are set out in our licence referred to above.

Other than as permitted in any relevant BMJ Author's Self Archiving Policies, I confirm this Work has not been accepted for publication elsewhere, is not being considered for publication elsewhere and does not duplicate material already published. I confirm all authors consent to publication of this Work and authorise the granting of this licence.

Confidential: For Review Only

Gastrostomy and congenital anomalies - a European population-based study

Ester Garne, Joachim Tan, Maria Loane, Silvia Baldacci, Elisa Ballardini, Joanna Brigden, Clara Cavero-Carbonell, Laura Garcia-Villodre, Mika Gissler, Joanne Given, Anna Heino, Sue Jordan, Elizabeth S Limb, Amanda Neville, Anke Rissmann, Michele Santoro, Ieuan Scanlon, Stine Kjaer Urhoj, Diana Wellesley, Joan Morris

- **Ester Garne**, ester.garne@rsyd.dk , Department of Paediatrics and Adolescent Medicine, Lillebaelt Hospital, University Hospital of Southern Denmark, Kolding, Denmark, ORCID 0000-0003-0430-2594
- **Joachim Tan**, jtan@sgul.ac.uk , Population Health Research Institute, St George's, University of London, UK, ORCID 0000-0003-0462-4761
- **Maria Loane**, ma.loane@ulster.ac.uk , Faculty of Life and Health Sciences, Ulster University, Northern Ireland, UK
- **Silvia Baldacci**, silviab@ifc.cnr.it , Unit of Epidemiology of Rare Diseases and Congenital Anomalies, Institute of Clinical Physiology, National Research Council, Pisa, Italy, ORCID 0000-0002-7626-1202
- **Elisa Ballardini**, MD, blllse@unife.it Neonatal Intensive Care Unit, Paediatric Section, IMER Registry (Emilia Romagna Registry of Birth Defects), Dep. of Medical Sciences, University of Ferrara, Italy. ORCID 0000-0002-8813-1835
- **Joanna Brigden**, jbrigden@sgul.ac.uk , Population Health Research Institute, St George's, University of London, UK
- **Clara Cavero-Carbonell,** cavero_cla@gva.es , Rare Diseases Research Unit, Foundation for the Promotion of Health and Biomedical Research in the Valencian Region, Valencia, Spain, ORCID 0000-0002-4858-6456
- **Laura García-Villodre,** garcia_lauvil@gva.es , Rare Diseases Research Unit, Foundation for the Promotion of Health and Biomedical Research in the Valencian Region, Valencia, Spain, ORCID 0000-0001-9094-7107
- **Mika Gissler,** mika.gissler@thl.fi , Department of Knowledge Brokers, THL Finnish Institute for Health and Welfare Helsinki, Finland; Region Stockholm, Academic Primary Health Care Centre, Stockholm, Sweden; Karolinska Institutet, Department of Molecular Medicine and Surgery, Stockholm, Sweden, ORCID 0000-0001-8254-7525
- **Joanne Given**, je.given@ulster.ac.uk , Faculty of Life and Health Sciences, Ulster University, Northern Ireland, UK, ORCID 0000-0003-4921-1944
- **Anna Heino**, anna.heino@thl.fi Department of Knowledge Brookers, THL Finnish Institute for Health and Welfare Helsinki, Finland
- **Sue Jordan,** s.e.jordan@swansea.ac.uk , Faculty of Medicine, Health and Life Sciences, Swansea University, Wales, UK, ORCID 0000-0002-5691-2987
- **Elizabeth S Limb**, elimb@sgul.ac.uk , Population Health Research Institute, St George's, University of London, UK, ORCID 0000-0003-0830-7376
- **Amanda J Neville**, Emila Romagna Registry of Birth Defects and Center for Clinical and Epidemiological Research, University of Ferrara, Azienda Ospedaliero- Universitaria di Ferrara, Corso Giovecca 203, 44121 Ferrara, Italy

Confidential: For Review Only

- **Anke Rissmann**, anke.rissmann@med.ovgu.de , Malformation Monitoring Centre Saxony-Anhalt, Medical Faculty Otto-von-Guericke-University Magdeburg, Magdeburg, Germany, ORCID 0000-0002-9437-2790
- **Michele Santoro**, msantoro@ifc.cnr.it , Unit of Epidemiology of Rare diseases and Congenital anomalies, Institute of Clinical Physiology, National Research Council, Pisa, Italy, ORCID 0000-0003-0676-3036
- **Ieuan Scanlon**, ieuan.scanlon@swansea.ac.uk , Faculty of Medicine, Health and Life Sciences, Swansea University, Wales, UK, ORCID 0000-0002-7479-5497
- **Stine Kjaer Urhoj**, stur@sund.ku.dk , Paediatric Department, Hospital Lillebaelt Kolding, Denmark and Section of Epidemiology, Department of Public Health, University of Copenhagen, Denmark. 0000-0002-2069-9723
- **Diana Wellesley**, dgw@soton.ac.uk , Clinical Genetics, University of Southampton and Wessex Clinical Genetics Service, Princess Anne Hospital, Southampton, UK, 0000-0002-9463-3188
- **Joan K Morris,** jmorris@sgul.ac.uk , Population Health Research Institute, St George's, University of London, UK, ORCID 0000-0002-7164-612X

Word count: 1938

Corresponding author

Ester Garne

Paediatric Department, Hospital Lillebaelt Kolding

Sygehusvej 24, DK-6000 Kolding

Ester.garne@rsyd.dk

Confidential: For Review Only

Abstract – 244 words

Objective. To report and compare the proportion of children with and without congenital anomalies undergoing gastrostomy for tube feeding in their first 5 years.

Methods. A European, population-based data-linkage cohort study (EUROlinkCAT). Children up to 5 years of age registered in nine EUROCAT registries (national and regional) in six countries and children without congenital anomalies (reference children) living in the same geographical areas were included. Data on hospitalisations and surgical procedures for all children were obtained by electronic linkage to hospital databases.

Results. The study included 91,504 EUROCAT children and 1,960,272 reference children. Overall, 1,200 (1.3%, 95% CI 1.2-1.6) EUROCAT children and 374 (0.016%, 95% CI 0.009-0.026) reference children had a surgical code for gastrostomy within the first 5 years of life. There were geographical variations across Europe with higher rates in Northern Europe compared to Southern Europe. Around one in four children with Cornelia de Lange syndrome and Wolf-Hirschhorn syndrome had a gastrostomy. Among children with structural anomalies those with esophageal atresia had the highest proportion of gastrostomy (15.9%).

Conclusions. This study including almost 2 million reference children in Europe found that only 0.016% of these children had a surgery code for gastrostomy before age 5 years. The children with congenital anomalies were on average 80 times more likely to need a gastrostomy before age 5 years than children without congenital anomalies. More than two thirds of gastrostomy procedures performed within the first 5 years of life were in children with congenital anomalies.

Confidential: For Review Only

- **What is already known on this topic:**

  - Gastrostomy for tube feeding is undertaken for children with major feeding problems and failure to thrive, but estimates of its prevalence are not readily available
  - The main indications for gastrostomy in children are severe neurological disorders and congenital anomalies with severe feeding problems

- **What this study adds:**

  - Only 16 per 100,000 children with no congenital anomalies have gastrostomy surgery within the first 5 years of life.
  - Children with congenital anomalies are on average 80 times more likely to need a gastrostomy before age 5 than children without congenital anomalies.
  - More than two-thirds of gastrostomy surgical procedures before age 5 are performed in children with major congenital anomalies.

- **How this study may affect research, practice or policy**

  - The existing heterogeneity in prevalence of surgery for gastrostomy in young children across Europe should be explored in future research.
  - Risks from this study of surgery for gastrostomy in children with specific congenital anomalies can be provided to their parents at the time of diagnosis.

Confidential: For Review Only

Introduction

Gastrostomy is used for infants and children with major, long-term feeding problems and failure to thrive[1]. The procedure of inserting a permanent gastrostomy is generally considered safe[2]. The main indications for a permanent or longterm gastrostomy are severe neurological disorders and congenital anomalies associated with severe feeding problems[3,4,5]. A 19% increase in the number of surgical procedures for gastrostomy on children over the years 1997-2009 has been reported using national data from US[6]. It has also been shown that, after surgery for gastrostomy, weight-for-length z-scores increase[7], as does the quality of life for the child and the caregiver[8]. A UK study found a point prevalence of gastrostomy in situ in January 2020 of 84 per 100,000 children for the age group 0-19 years and 79.6 per 100,000 for the younger age group 0-4 years[5]. Little is known about the frequency of gastrostomy in children with specific congenital anomalies that are associated with feeding problems and failure to thrive.

The aims of this EUROlinkCAT study were to report 1) the frequency of surgery for gastrostomy in children with congenital anomalies up to the age of 5 years compared with the general population of all children without congenital anomalies living in the same geographical areas and 2) the prevalence of gastrostomy in children with specific congenital anomalies.

Methods

This is a European, population-based data-linkage cohort study using routinely collected health service data. The study includes data from nine European surveillance of congenital anomalies (EUROCAT) registries (national and regional) in six countries, see Table 1. Children with major congenital anomalies as defined by EUROCAT[9] born between 1995 (or the first year of the EUROCAT registry if later) and 2014 were included (EUROCAT children). For five registries data on all live born children without congenital anomalies from the same population covered by the registry and the same birth years were included as the reference population (reference children). The Tuscany registry used a 10% random sample of their population as the reference children. There were no reference children available from the three English registries.

Data on hospitalisations and surgical procedures for all children up to the child's 10th birthday or end of 2015, whichever came earlier, were obtained by electronic linkage to the hospital databases used in the regions and countries. This ensured that at least one year of follow-up after birth was available for each

Confidential: For Review Only

child. We included children up to age of 5 years of age in this study as the number of children reaching the age of 10 years with the specific congenital anomalies under consideration was limited. Details of the methods used in the EUROlinkCAT study including the linkage methods have been published elsewhere[10,11].

The hospital databases in Finland, Denmark (Funen), Tuscany in Italy and England (East Midlands & South Yorkshire, Thames Valley and Wessex) covered hospitalisations of the study population in the whole country. For Wales, this included procedures carried out in England. For Valencian Region in Spain and Emilia Romagna in Italy, the hospital databases covered the same region as the EUROCAT registry.

Surgical procedures were coded according to the coding systems used in the national health systems. Italy and Spain used ICD-9-CM (the International Classification of Diseases, 9th revision - Clinical Modification) for the study period, England and Wales used OPCS-4 (Classification of Interventions and Procedures) and Finland and Denmark used national adaptions of NCSP (NOMESCO Classification of Surgical Procedures). The surgery codes used for gastrostomy were 43.11 and 43.19 in ICD-9-CM, G341 and G342 in OPCS-4.8 and JDB in NCSP.

Data were analysed for all children with major congenital anomalies and additional analyses performed on children with the following specific anomalies: spina bifida, hydrocephaly, congenital heart defects and severe congenital heart defects, cleft lip with and without cleft palate, cleft palate, esophageal atresia, Down syndrome, Goldenhar association, Di George syndrome, Noonan syndrome, Williams syndrome, Cri du chat syndrome, Wolf-Hirschhorn syndrome, Angelmann syndrome and Cornelia de Lange syndrome. These anomalies all had specified ICD-9 and ICD-10 codes in the EUROCAT database and were selected from the EUROlinkCAT list of anomaly subgroups [10] by a paediatrician (EG) as associated with feeding problems.

There was no parent or public involvement in the study.

Statistical methods.

Each registry standardised their data using the EUROlinkCAT common data model which enabled them to run a centrally written analysis script on their individual case data. The proportions of children receiving a surgical gastrostomy code before the age of 5 years was calculated using Kaplan Meier Survival Analysis (KM) to account for the censoring occurring on 1st January 2016. This enabled children who were born between 2010-2014 and who did not reach age 5 years to be included in the analysis. Each registry submitted the numbers of births, the numbers of children with a gastrostomy code before the age of 5 years and the KM estimates, by type of congenital anomaly, to the central results repository at Ulster University via a secure portal (no individual case data were provided to Ulster University). These results for

Confidential: For Review Only

all registries were then provided to the researchers via the same portal. The confidence intervals for the KM survival analysis estimates were calculated by STATA using the ln(-ln(S(t))) transformation. To obtain pooled estimates of the percentage of children with surgical gastrostomy code across registries random effects inverse-variance meta-analyses were performed using the ln(-ln(S(t))) transformation. For several anomalies where no children in a single registry received a surgical gastrostomy code the upper 95% confidence limit (CI) was calculated using the exact binomial estimate, the percentage of children having gastrostomy was estimated to be 0.001% and the lower 95% CI was calculated assuming symmetry on the ln(-ln(S(t)) scale. The meta-analysis was performed in Stata (version 15).

Ethical approval

All EUROCAT registries obtained ethical, governance and other permissions for the data linkage according to their national legislations and arrangements. University of Ulster obtained Ethics permission for the Central Results Repository on 15.09.2017 (Institute of Nursing and Health Research Ethics Filter Committee, number FCNUR-17- 000)

Results

The study included 91,504 EUROCAT children with major congenital anomalies and 1,960,272 reference children. Overall, 1,200 (1.3%, 95% CI 1.2-1.6) EUROCAT children and 374 (0.016%, 95% CI 0.009-0.026) reference children had a surgery code for gastrostomy within the first 5 years of life. There were geographical differences across Europe with higher rates in the Nordic countries and UK (1.3-2.1% of EUROCAT children) and lower rates in Italy and Spain (0.8-1.0 % of EUROCAT children). In the five registries with reference children for the whole population (Funen, Finland, Wales, Emilia Romagna and Valencian Region) there were 883 EUROCAT and 374 reference children with a surgery code for gastrostomy, in other words 70% of all gastrostomies within the first 5 years were performed on children with congenital anomalies.

Table 2 describes the number of children with specific congenital anomalies and the proportions with surgery codes for gastrostomy. Around one in four of the children with Wolf-Hirschhorn syndrome or Cornelia de Lange syndrome had a surgery code for gastrostomy before the age of 5, although the 95% CIs are wide due to the small number of children diagnosed with these rare syndromes. For children with esophageal atresia the proportion was 15.9% (95% CI 13.5 – 18.5). The larger group of children with congenital heart defects had a lower prevalence of surgery codes for gastrostomy (0.5%, 95% CI 0.4-0.7) and for children with severe congenital heart defects the proportion was 1.3% (95% CI 0.8-2.0). For children

Confidential: For Review Only

with facial clefts the percentage with a surgery code for gastrostomy was highest for children with cleft palate (4.1%, 95% CI 3.4-4.9).

Discussion

This study with European population-based data showed that surgery for gastrostomy within the first five years of life was on average 80 times more frequent in children with congenital anomalies than in children without congenital anomalies from the same geographical areas. The study also showed a difference between Northern and Southern Europe: children with CAs were overall twice as likely to receive gastrostomy in the Nordic countries and UK when compared with Italy and Spain; this could be due in part to the regional coverage of hospital databases in Valencian Region and Emilia Romagna. There are limited data published on the frequency of gastrostomy in children with specific congenital anomalies and this study contributes to this literature.

A study from Germany showed that 24% of newborns in 2009-2013 with esophageal atresia had a gastrostomy during their neonatal hospital stay[12]. This is higher than the 15.9% found in our study, where the proportion was a calculated average for nine regions in Europe and also included children born in an earlier time period.

The proportion of children with congenital heart defects having surgery for gastrostomy was low at 0.5%, but the risk was still 30 times higher than in the reference population (0.016%). For children with severe congenital heart defects, gastrostomy for feeding after neonatal cardiac surgery improves growth velocity in their first year of life[13].

Despite the multicenter dataset case numbers were too small to analyse by registry and over time. A study analysing data over time in the whole paediatric population in the US showed that the number of procedures for gastrostomy increased every year from 1997 to 2009[6]. Also studies from Australia and Sweden have shown increase over time in the insertion and use of permanent feeding tubes in the paediatric population[14,15]. It is likely that surgeries for gastrostomy also increased in the geographical areas included in our study during the 20 years study period. A European study on children with cerebral palsy also found a difference in prevalence of gastrostomy across Europe with the highest prevalence in Sweden and the lowest prevalence in Portugal[16]. The study also found a low prevalence in Northern England. Our study did not find a low prevalence of gastrostomy in the English registries. Furthermore, our study cannot say why there are differences in the use of gastrostomy. These differences may be cultural in

Confidential: For Review Only

nature, with different levels of acceptance by parents, or regional or national, such as access to the surgery and doctors' practices when discussing the surgery for gastrostomy with parents.

We do not have data on the duration of the tube feeding through the gastrostomy and if the gastrostomy was removed as soon as the feeding problems were resolved. It should be acknowledged that both the presence of feeding problems at a certain age and the surgery for gastrostomy to treat them, will increase the stress for the child and the family[17].

The main strength of this study is that the population-based data included all children with major congenital anomalies and not only those referred to tertiary hospitals. Further, the EUROCAT registries have high levels of case ascertainment and use standardised definitions and coding of congenital anomalies to ensure consistency across Europe. The data in the hospital databases were also standardised as part of the EUROlinkCAT study before analysis scripts were written and distributed. The study also has limitations. The percentage of children with a surgery code for gastrostomy may be higher than presented here as the surgery codes may not always be registered in the hospital databases and variations in coding practices may mean that some relevant codes were not identified. There may be problems with the registration of procedures before the newborns have their permanent name or ID number allowing them to be matched to their surgery data, and some children may have been referred for surgery outside the area covered by the hospital databases in this study (although this affected only two registries). All of these would lead to an underestimation of the prevalence of gastrostomy in the study population.

Conclusions

This study including almost 2 million reference children in Europe found that only 0.016% of these children had a surgery code for gastrostomy before age 5 years. Thechildren with congenital anomalies were on average 80 times more likely to need a gastrostomy before age 5 years than the children without congenital anomalies. This is important information when counseling the parents about the expected morbidity for their child after a prenatal or postnatal diagnosis of a specific congenital anomaly associated with feeding problems.

Confidential: For Review Only

Funding

This project has received funding from the European Union's Horizon 2020 research and innovation programme under grant agreement No. 733001. The funders had no role in the design and conduct of the study; collection, management, analysis, and interpretation of the data; preparation, review, or approval of the manuscript; and decision to submit the manuscript for publication.

Competing interest declaration:

All authors declare no financial relationships with any organisations that might have an interest in the submitted work in the previous three years; no other relationships or activities that could appear to have influenced the submitted work.

Contributors

JM, ML and EG designed the EUROlinkCAT study and obtained funding. CC, MG, HdeW, AN, DW and EG contributed with data from their EUROCAT registries. SKU, JT, AH, MS, LG, SB, EB, CC, lS, SJ and JB linked the EUROCAT data to the hospital databases. ML and JG performed the standardization of the variables in collaboration with the local registries. AR and EG defined the surgical variables. JM and EL performed the statistical analysis. EG wrote the manuscript. All authors reviewed and revised the manuscript.

Confidential: For Review Only

References

1. Frölich T, Richter M, Carbon R, Barth B, Köhler H. Review article: percutaneous endoscopic gastrostomy in infants and children. Aliment Pharmacol Ther 2010; 31:788-801

2. Kumbhar SS , Plunk MR , Nikam R, Boyd KP, Thakrar PD. Complications of percutaneous gastrostomy and gastrojejunostomy tubes in children. Pediatr Radiol 2020;50(3):404-414.

3. Wiechers C, Thjen T, Koos B, Reinert S, Poets CF. Treatment of infants with craniofacial malformations. Arch Dis Child Fetal Neonatal Ed 2021; 106:104-109

4. Fleming H, Dempsey AG, Palmer C, Dempsey J, Friedman S, Galan HL, Given j. Primary contributors to gastrostomy placement in infants with congenital diaphragmatic hernia. J Pediatr Surg 2021; 56:1949-1956

5. Pardy C, Kharma N, Lau R, Kelly V, Yardley I. Point prevalence of gastrostomy in a paediatric population. JPGN 2021; 72:528-31

6. Fox D, Campagna EJ, Friedlander J, Partrick da, Rees DI, Kempe A. National trends and outcomes of pediatric gastrostomy tube placement. JPGN 2014; 59:582-88

7. Franken J, Mauritz FA, Suksamanapun N, Hulsker CCC, van der Zee DC, van Herwaarden-Lindeboom MYA. Efficacy and adverse events of laparoscopic gastrostomy placement in children: results of a large cohort study. Surg Endosc 2015; 29:1545-52

8. Pemberton J, Frankfurter C, Bailey K, Jones L, Walton JM. Gastrostomy matters – the impact of pediatric surgery on caregiver quality of life. J Pediatr Surg 2013; 48:963-70

9. Kinsner-Ovaskainen A, Lanzoni M, Garne E, et al. A sustainable solution for the activities of the European network for surveillance of congenital anomalies: EUROCAT as part of the EU Platform on Rare Diseases Registration. European journal of Medical Genetics 2018; 61: 513-7

10. Morris JK, Garne E, Loane M et al. EUROlinkCAT protocol for a European population-based data linkage study investigating the survival, morbidity and education of children with congenital anomalies. BMJ Open 2021;11(6):e047859

11. Loane M, Given JE, Tan J et al. Linking a European cohort of children born with congenital anomalies to vital statistics and mortality records: A EUROlinkCAT study. PLOS ONE. 2021;16(8):e0256535. doi: 10.1371/journal.pone.0256535.

12. Schmedding A, Wittekind B, Schloesser R , Hutter M , Rolle U. Outcome of esophageal atresia in Germany. Dis Esophagus 2021;34(4):doaa093

13. Sochet AA, Grindy AK, Son S, Barrie EK, Hickok RL, Nakagawa TA, Wilsey MJ. Percoutaneous Endoscopic Gastrostomy after Cardiothoracic Surgery in Children less than 2 months old: An assessment of long-term malnutrition status and gastrostomy outcomes. Pediatr Crit Care Med 2020; 21:50-58

14. Wong K, Leonard H, Pearson G et al. Epidemiology of gastrostomy insertion for children and adolescents with intellectual disability. Eur J Pediatr 2019; 178:351-61

15. Backman e and Sjögreen L. Gastrostomy tube insertion in children with developmental or acquired disorders: a register-based study. Dev Med Child Neurol 2020; 62: 1191-7

16. Dahlseng M, Andersen GL, Graca Andrada MDA, Arnaud C, Balu R, De la Cruz J, Folha T, Himmelmann K, Horridge K, Júlíusson PB, Påhlman M, Rackauskaite G, Sigurdardottir S, Uldall P, Vik T, Surveillance of Cerebral Palsy in Europe Network. Gasrostomy tube feeding of children with cerebral palsy: variation across six European countries. Dev Med Child Neurol 2012; 54:938-44

17. Silverman AH, Erato G, Goday P. The relationship between chronic paediatric feeding disorders and caregivers stress. J Child Health Care 2021; 25:69-80

Confidential: For Review Only

Table 1. Number of children with and without congenital anomalies and proportions with a surgery code for gastrostomy before age 5 years, by registry

	EUROCAT children			Reference children		
	Number of children included in study	Children with surgery for a feeding tube age 0-4 years		Number of children included in study	Children with surgery for a feeding tube age 0-4 years	
		Number	Percentage (95% CI)		Number	Percentage (95% CI)
Denmark, Funen 1995-2014	2423	40	1.8 (1.3-2.4)	100748	33	0.03 (0.02-0.05)
Finland 1997-2014	38324	552	1.5 (1.4-1.7)	911679	255	0.03 (0.03-0.03)
Italy, Tuscany 2005-2014	4225	35	0.9 (0.6-1.2)	23503[1]	0	0.00 (.-.)
Italy, Emilia Romagna 2008-2014	5381	51	1.0 (0.8-1.4)	223995	21	0.01 (0.01-0.02)
Spain, Valencian Region 2010-2014	4260	30	0.8 (0.6-1.2)	168563	12	0.01 (0.00-0.01)
UK, Wales 1998-2014	17448	210	1.3 (1.2-1.5)	531784	53	0.01 (0.01-0.02)
UK, Thames Valley 2005-2013	3845	46	1.3 (1.0-1.8)	n/a		
UK, E Midlands and S Yorkshire 2003-2012	11278	158	1.6 (1.3-1.8)	n/a		
UK, Wessex 2004-2014	4320	78	2.1(1.7-2.6)	n/a		
Total	91504	1200	1.3 (1.2-1.6)[2]	1960272	374	0.016 (0.009 - 0.026)[2]

[1]Reference children Tuscany 10% of population

[2]Estimated percentages and 95% confidence intervals are calculated using a random effects meta-analysis of individual registries' estimates and not from the total numbers given in the final row of the table.

Confidential: For Review Only

Table 2. Meta-analysis of proportion of children with specific congenital anomalies having a surgery code for gastrostomy before 5 years of age.

Anomaly	Number of liveborn children	Children with a surgery for gastrostomy at age 0-4 years (adjusted for censoring)	
		Number	Percentage (95% CI)
Spina bifida	591	11	2.1 (1.0-3.7)
Hydrocephaly	1082	52	5.5 (3.3-8.4)
Congenital heart defects	26442	125	0.5 (0.4-0.7)
Severe Congenital heart defects	5693	72	1.3 (0.8-2.0)
Cleft lip with or without cleft palate	2961	37	1.4 (1.0-2.0)
Cleft palate	2879	109	4.1 (3.4-4.9)
Esophageal atresia	915	138	15.9 (13.5-18.5)
Down syndrome	3640	62	1.1 (0.5-2.2)
Goldenhar association	301	27	9.7 (6.5-13.6)
Di George syndrome	292	25	7.8 (3.3-14.9)
Noonan syndrome	154	12	8.2 (3.6-15.1)
William syndrome	115	4	2.9 (0.4-10.7)
Cri du chat syndrome	57	8	15.8 (6.0-29.8)
Wolf-Hirschhorn syndrome	37	8	23.9 (3.4-54.6)
Angelmann syndrome	44	2	3.3 (0.2-15.9)
Cornelia de Lange syndrome	34	9	29.2 (11.8-49.2)

Confidential: For Review Only
